# Read to the Muskingum Co. Med. Society

**Published:** 1870-12

**Authors:** A. Ball


ARTICLE III.—*Read to the Muskingum County Medical Society.* By A. BALL, M.D.

The case reported in the CHICAGO MEDICAL JOURNAL, by Dr. McElroy, entitled "Clinical Experience in Private Practice, from a Physical Basis of Life," requires some corrections and some comments.

Being called in council with Dr. McElroy, on April 5th, and seeing the boy only twice, that morning with Dr. M., and the same afternoon, at his request, in his absence, I can only speak of facts as they occurred under my observation. About 8 o'clock that morning Dr. M. called at my office, saying he had a very singular case in young Patterson. He had visited him the day before and again that morning, and found him getting worse. He asked me to see the case with him, stating that he had called before for me, and regretted that I was absent, as he thought if ammonia had been injected into his veins, then there would have been some hope of saving the patient; and, as it was a case presenting no hope under any other treatment, the experiment would be perfectly justifiable. "But now," he remarked, "I fear it is too late to do any good."

On arriving at the house, we found the boy throwing himself about the bed in an unconscious state. His surface was cold and livid. Froth was working from his mouth, and he was pulseless at the wrist. Dr. M. examined his pulse first at the wrist, saying there was no pulse at that point. He then felt in the axilla, and called my attention to the fact that there was a feeble pulse in that location. I then examined carefully, and fully corroborated his statement: finding no pulse at the wrist, none at the bend of the elbow, but a feeble, fluttering pulse in the axilla. I have no recollection of either of us attempting to count it; the pulse being so feeble, and the boy so restless, it would probably have been fruitless to do so. Placing my hand on the chest, I found a *rough*, *harsh* respiration, interfering, by the accumulation of partially dried mucus in the small bronchi, with the respiration, owing to the loss of sensibility, and physical prostration.

On retiring to consult, I was not a little astonished and alarmed on learning that the boy had taken *three ten-drop doses of Veratrum Viride in as many hours*. My first thought was how to counteract the effect of the Veratrum, which had been taken in what I considered such unjustifiable and dangerous doses. We had no difficulty in agreeing upon the treatment, which was brandy and milk, and hydrate of chloral, in about seven-grain doses, every hour until rest, and a jacket of mustard poultice over the entire chest. Dr. M. was compelled to be absent through the day, and requested me to see the boy at noon. I saw him at two o'clock P. M., and was informed that *he had neither vomited nor purged* between my first and my second visit. But, under the change of treatment, he had rapidly become quiet. He had perfectly reacted. His surface was florid. His pulse was active, *even feverish.* The breathing was full and easy, and the boy seemed partially conscious. I at once ordered the stimulants stopped, and directed a resumption of Veratrum in *two-drop* doses every two hours, until the fever should subside. I did not see him again during his sickness, but was informed daily of his improvement.

I have reported the *facts* thus in detail, because in some important particulars they differ from the statements made by Dr. M., and also that each may judge for himself *what remedies promoted and what retarded the recovery* of the boy, without reference to whether they *sustain* or *condemn* the treatment "from a physical basis of life." With all deference to Dr. M.'s opinion of the case, as to the remedies which were beneficial, I am constrained to regard the depression as largely due to the veratrum; and but for the prompt use of the brandy and the chloral, we would have had a fatal termination. I hope the facts in this case will be a warning to others to avoid the reckless use of so potent and so dangerous a remedy as that upon which Dr. M. so much relies. I am at a loss to see how an impartial seeker after true therapeutic effects can regard veratrum as other than a *hindrance* in this case. It shows that when a theory is adopted by one as enthusiastic as my friend, he can only see results of certain treatment as he may imagine them to comport with his theory. It will be seen by the above that Dr. M. *had no hope of his case under the veratrum treatment*, and considered it too far gone for his ammonia experiment, when he adopted the brandy and chloral treatment. It will also be seen that *he had forgotten the facts* when he stated that the pulse was perceptible at the wrists at the time we were in council. He is further and *materially* in error in stating that there was vomiting and purging *after the chloral and brandy* were given, as *neither* occurred between my visit in the morning and that in the afternoon. And, as to the chloral being "inert," it was followed by the *only result hoped for* when we ordered it, viz., rest and reaction. And its result was much more prompt and efficient than either of us anticipated. Permit me to say that I regard the case as one of a wonderful escape from overdosing with veratrum, and but for the "little jar due to the consultation," the boy would now be numbered with the dead. Indeed, I think the result would justly authorize us to classify chloral hydrate as one of the antidotes to veratrum in overdoses.

To review the facts: the boy was found in an insensible state, pulseless and prostrate. Three fourths of an ounce of chlorate of potash is given during the night. He is pulseless and prostrate in the morning. Tincture of Veratrum Viride is given at 5 o'clock in the morning. Dose 10 drops, which is repeated at 6 and 7 o'clock. We visit the patient at 8 and find him worse, more restless and more prostrate. He has vomited and purged unconsciously in bed; is pulseless at the wrist, and all are of the opinion that he is dying. In this condition we find the patient, when brandy and milk and chloral are ordered, together with a mustard poultice enveloping the whole chest. Everything else is stopped. The vomiting and purging cease. The restlessness soon subsides. The surface becomes florid; the pulse is fully restored at the wrist, and consciousness is returning. Now let me ask any reasonable man what remedies gave the boy relief. I will not attempt to insult your intelligence or judgment by suggesting an answer.

—

[ZANESVILLE, NOV. 2, 1870.

*Messrs. Editors:* It was agreed by the Society, that the remarks of the several fellows, on the reported case of Drs. McElroy and Ball, should be published in the JOURNAL, but the Secretary did not note the remarks at the time, nor have the members who participated at the time furnished him such. Suffice it to say, that Drs. Culbertson, Bell, Hildreth, and Prof. T. A. Reany, spoke condemnatory of the treatment, as given by Dr. McElroy.

J. R. LARZELERE, Secy.]

